# Low uptake of hepatitis B vaccination among healthcare workers in primary health facilities in Mwanza region, North-Western Tanzania

**DOI:** 10.3389/fpubh.2023.1152193

**Published:** 2023-06-02

**Authors:** Bernada Ndunguru, Diana Wilfred, Anthony Kapesa, Semvua D. Kilonzo, Mariam Mirambo, Fred Hyera, Fabian Massaga

**Affiliations:** ^1^School of Public Health, The Catholic University of Health and Allied Sciences-Bugando, Mwanza, Tanzania; ^2^Department of Pediatrics and Child Health, Bugando Teaching and Consultant Hospital, Mwanza, Tanzania; ^3^Department of Community Medicine, School of Public Health, The Catholic University of Health and Allied Sciences-Bugando, Mwanza, Tanzania; ^4^Department of Internal Medicine, The Catholic University of Health and Allied Sciences-Bugando, Mwanza, Tanzania; ^5^Department of Microbiology and Immunology, The Catholic University of Health and Allied Sciences- Bugando, Mwanza, Tanzania; ^6^Department of Research and Consultancy, Bugando Teaching and Consultant Hospital, Mwanza, Tanzania; ^7^Department of General Surgery, Bugando Teaching and Consultant Hospital, Directorate of Surgical Services, Mwanza, Tanzania

**Keywords:** coverage of hepatitis B vaccination, hepatitis B virus, healthcare workers, primary health facilities, uptake

## Abstract

**Background:**

Despite the availability of hepatitis B vaccines (HBV) in Tanzania, their uptake among healthcare workers (HCWs) in high-level facilities, such as tertiary hospitals where the vaccines are available, is low. However, their uptake among HCWs in primary health facilities remains understudied. The lack of this information limits the scaling up of HBV vaccination programs.

**Methodology:**

A cross-sectional analytical study was conducted between June and July 2022 among HCWs in the Misungwi and Ilemela districts, which were purposefully selected. The sample size was calculated using the Taro Yamane formula, and data were collected using a self-administered questionnaire and analyzed using IBM SPSS^®^ version 25.

**Results:**

A total of 402 HCWs were recruited, their mean age was 34.9 ± 7.77 years, and only 18% (76/402) reported being fully vaccinated. HCWs in Ilemela showed higher uptake (*χ*^2^ = 23.64, df = 1, *p* = 0.00) of the vaccine than HCWs in Misungwi. Being male (aOR = 2.38, 95% CI 1.28–4.45, *p* = 0.006), working in an urban setting (aOR = 5.75, 95% CI 2.91–11.35, p = 0.00), and having an employment duration of more than 2 years (aOR = 3.58, 95%CI 1.19–10.74, *p* = 0.023) were significantly associated with higher odds of vaccination. Moreover, high perceived susceptibility to HBV infection (aOR = 2.20, 95% CI1.02–4.75, *p* = 0.044) and history of needle prick injuries (aOR = 6.87, 95%CI 3.55–13.26, *p* = 0.00) were significantly associated with higher odds of HBV vaccination.

**Conclusion:**

Low uptake of HBV vaccine among HCWs in primary health facilities was observed with a noteworthy difference between rural and urban settings. Therefore, advocacy campaigns and resource mobilization toward the promotion of HBV vaccination in primary health facilities are pivotal.

## 1. Introduction

Hepatitis B viral infection (HBV) is a deadly blood-borne vaccine-preventable liver disease affecting more than 2 billion people worldwide (1). It is the top cause of liver cirrhosis and hepatocellular carcinoma (2, 3). Globally, the prevalence of HBV infections is estimated to be around 1.3%, with rates ranging from as low as 0.2% in the United States of America to as high as 8% in Africa (4). In Tanzania, the prevalence of HBV was reported to range from 5.5 to 20% in the general population (3).

Healthcare workers (HCWs) are four times more likely to acquire HBV infection than the general population. This is due to the fact that they encounter many occupational risk exposures such as needle stick injuries and body fluid splashes, among others (5, 6). According to a global policy report on the prevention and control of viral hepatitis among WHO member states, approximately 2 million HCWs are at risk of being infected with HBV each year in a midlist of low vaccination coverage (2). A recent meta-analysis study conducted in Africa and Asia reported the prevalence was reported to be 4.0% and 5.0% in Asia and Africa respectively (7). In Tanzania, the prevalence of HBV infection among HCWs is even higher, ranging from 5.7 to 7% (8, 9).

To prevent HBV infection among HCWs, various interventions have been recommended. These include but are not limited to observing infection control strategies, blood safety through screening before transfusion, and vaccination. Of these, the latter is the most cost-effective intervention, as it conveys a protective efficacy of more than 90 percent (10).

Occupational HBV infections have been reported to affect about 37% of HCWs worldwide (11). In the United States of America, a study conducted between 2002 and 2003 by Somard et al. showed a good response toward HBV vaccination, where 75% of the HCWs had received three doses of the HBV vaccine, meaning that they were fully vaccinated against HBV (12). The coverage of HBV vaccination showed to be higher among nurses and doctors as compared to other HCWs. Another study conducted in Italy in 2019 showed that almost all HCWs had received the HBV vaccination (13). On the other hand, there are limited data in low-middle-income countries (LMICs) on the vaccination coverage among HCWs; however, the available data are useful enough to understand the situation of HBV vaccination status. In LMICs, it is reported that 50% of HBV infections are occupationally related, and there is still low vaccination coverage among HCWs. For instance, a study conducted in Egypt in 2003 found that only 15.8% of HCWs had received three doses of the HBV vaccine. In Tanzania, despite the availability of the HBV vaccine, only one out of five HCWs in tertiary referral hospitals had protective immunity resulting from active vaccination (8).

Factors that have been found to influence vaccination against HBV can be grouped as individual or health system factors. The individual factors that have been found to affect the uptake of the HBV vaccine among HCWs include knowledge and awareness, perceptions, distrust in vaccines, and cost. Health system factors found to affect HBV vaccine uptake include the vaccine availability at a facility followed by a lack of information on where to get the vaccine (14).

Despite the availability of hepatitis B vaccines (HBV) in Tanzania, their uptake among healthcare workers (HCWs) in tertiary hospitals where the vaccines are available is still low, as uncovered in the studies (8, 9). On the other hand, their uptake among HCWs in primary health facilities remains understudied. The lack of this information limits the implementation and scaling up of HBV vaccination programs in these facilities. Therefore, this study aimed to explore the magnitude of HBV vaccination coverage among HCWs in primary health facilities and the factors that influence vaccination among them.

## 2. Materials and methods

### 2.1. Study area, design, and participants

This was a cross-sectional analytical study conducted in the Misungwi and Ilemela districts, both located in the Mwanza Region. A total of 339 and 473 HCWs were providing health services in primary health facilities in Ilemela and Misungwi, respectively, during the time of the study. The former district is located in an urban area, whereas the latter is in a rural setting. There are 14 dispensaries in the Ilemela district and 4 public health centers, whereas the Misungwi district has a total of 43 dispensaries and 4 health centers.

### 2.2. Sample size estimation and sampling techniques

The sample size was calculated by using the Taro Yamane formula (15) to give the minimum sample size required. The obtained sample size was multiplied by 1.5 to cover for the design effect, and the sample size of 402 participants was subsequently reached. The two study areas were purposefully selected to represent rural and urban settings. To ensure representativeness, two-thirds of all primary health facilities from each district were randomly selected. A total of 185 and 217 participants were selected to take part in Misungwi and Ilemela districts, respectively. HCWs from nine dispensaries and two health centers were selected in the Ilemela district, whereas HCWs from two health centers and 14 dispensaries from the Misungwi district were randomly selected from both medical and non-medical personnel.

### 2.3. Eligibility criteria

#### 2.3.1. Inclusion criteria

The study participants were HCWs working at the selected primary health facilities and had served in that particular facility for more than 1 year.

#### 2.3.2. Exclusion criteria

HCWs born after 2002 were excluded from the study, as they are more likely to have been vaccinated by the national expanded program for immunization.

### 2.4. Data collection, tools, and procedures

A pre-tested, semi-structured, self-administered questionnaire was used for data collection. The questionnaire had a total of seven sections. The sections included socio-demographic information of the participants, exposure status to HBV and its risk factors, awareness of HBV, knowledge of the participants on HBV, HBV vaccination status, and their perception toward HBV and vaccines. Awareness was measured using a single question that required a participant to answer whether they had heard of HBV before or not. Knowledge assessment was performed by adapting a work from Abdul Hakeem et al. (16). In this section, participants were asked a total of 10 questions on HBV transmission, prevention, and treatment to obtain an impression of their understanding of the disease. HBV vaccine uptake was determined by a section in the questionnaire where the participants were asked whether they had been vaccinated or not. Then, they were asked about the number of HBV vaccine shots they had received. Participants who had received a total of three shots were regarded as vaccinated, and those who had received less than three shots or none were considered not vaccinated. Perception of HBV vaccine and infection was measured using the six constructs of the health belief model, namely, perceived severity, perceived susceptibility, perceived benefits, perceived self-efficacy, perceived barriers, and cues to action, where each of the constructs had several statements (17). The statements were in the form of questions, with a minimum of two statements on the construct.

### 2.5. Data analysis

Data were entered into an Excel^®^ sheet and then exported to IBM SPSS^®^ version 25 for analysis. The continuous variables were summarized as the mean with standard deviation or median with the interquartile range depending on the distribution. The categorical variables were summarized using frequencies and proportions. For the identification of factors associated with the uptake of HBV vaccination, bivariate logistic regression was performed for each independent variable. Variables with value of ps lower than 0.05 were considered statistically significant. Multivariate analysis was performed using a multivariate logistic regression model for all variables with value of ps ≤0.2 during the bivariate analysis.

Knowledge assessment was measured using the following questions in the knowledge section of the questionnaire: How is HBV transmitted? Is HBV a preventable disease? What can be done to prevent HBV infection? If a person is found to be HBV positive, what will be done in Tanzania? Is HBV DNA recombinant vaccine capable of protecting a vaccinated person against HBV infection? Who is at risk of being infected with HBV? Is the HBV vaccine available in Tanzania? What is the minimum number of HBV shots needed to protect against HBV? Is it important to conduct an immune response test after the HBV vaccine? What is to be done after being accidentally in contact with an HBV-infected person’s blood or body fluid products? What is the route of the HBV recombinant vaccine? Each correct response scored one mark while the wrong response and I do not know response scored zero. The total scores for each participant were obtained and converted into percentages, and they were categorized as follows: poor knowledge <50%, fair knowledge 50–74%, and good knowledge ≥75% (16).

Perception of HBV vaccination was categorized as good perception and poor perception. The variables were recoded into different variables, and for each of the constructs of the health belief model, a set of questions was asked with the response options of agree, not sure, and disagree. These were subsequently given a score of 3, 2, and 1 point, respectively. Participants with a score above the median on each construct of the health belief model were considered to have a good perception of HBV vaccination, and those with a score below or equal to the median were considered to have a poor perception.

## 3. Results

### 3.1. Socio-demographic characteristics of study participants

This study sampled 402 HCWs from 27 health facilities in the Misungwi and Ilemela districts. The mean age of the participants was 34.9 ± 7.7 years (95% CI, 34.1–35.1), and the majority were female 56.5% (227/402). A total of 217 (54%) HCWs were recruited from Misungwi and the rest from the Ilemela district. Nurses were the majority 36.8% (148/402) of all medical cadres who participated in the study. Further details on the above descriptions are tabulated below (Table 1).

**Table 1:** Socio-demographic characteristics of study participants (*N* = 402).

Variable	Frequency (*n*)	Percentages (%)
Sex		
Male	175	43.5
Female	227	56.5
Age groups		
21–24 years	7	1.7
25-44 years	342	85.1
45-64 years	53	13.2
Districts		
Ilemela	185	46.0
Misungwi	217	54.0
Level of health facility		
Dispensary	164	40.8
Health center	238	59.2
Religion		
Christian	336	83.6
Muslim	68	16.4
Marital status		
Widowed	15	3.7
Divorced	3	7.0
Cohabiting	35	8.7
Single	90	22.4
Married	259	54.4
Education level		
Secondary education	10	2.5
Bachelor’s degree	43	10.7
Diploma	154	38.3
Certificate	195	48.5
Medical cadre		
Pharmacist	12	3.0
Medical doctor	33	8.2
Laboratory personnel	44	10.9
Medical attendant	79	19.7
Clinical officer	86	21.4
Nurses	148	36.8
Duration of employment		
1 year	14	3.5
2 years	37	9.2
3 years	39	9.7
4 years	27	6.7
More than 4 years	285	70.9
Departments served		
In patients	219	54.5
Outpatients clinic	92	22.9
Laboratory	59	14.7
Reproductive and child health	155	38.6
Theater	63	15.7
Labor ward	169	42.0
Mortuary	11	2.7

### 3.2. Risk exposures of HBV infection among HCWs in Misungwi and Ilemela districts

Nearly half 46.3% (186/402) of the participants reported having tested for HBV infection, and they all reported a negative status. Additionally, a total of 188 (46.8%) HCWs were reported to have been exposed to needle prick injury in the past year before the study. On the other hand, 90.3% of all the study participants used protective gear when attending to their clients. Table 2 shows hepatitis B risk exposures and protective behavior assessment among the study participants.

**Table 2:** Exposure to the risk of HBV infection among HCWs in Misungwi and Ilemela districts (*N* = 402).

Variable	Frequency (*n*)	Percent (%)
Ever tested for HBV		
Yes	186	46.3
No	216	53.7
HBV status		
Positive	0	0
Negative	186	100
Attended HBV-positive patient		
Yes	170	42.3
No	232	57.7
Needle prick injury in the past year		
Yes	188	46.8
No	214	53.2
How many needle picks in 1 year		
One	105	26.1
Two	48	11.9
Three	22	5.5
Four	11	2.7
More than four	2	0.5
Body fluid splashes from patients		
Yes	334	83.1
No	68	16.9
History of blood transfusion		
Yes	28	7.0
No	374	93.0
History of surgeries		
Yes	99	24.6
No	303	75.4
History of dialysis		
Yes	15	3.7
No	387	96.3
History of invasive procedures such as IV, IM, and endoscopy		
Yes	277	68.9
No	125	31.1
Number of sexual partners		
One	340	84.6
Two	50	12.4
Three	4	1.0
More than three	8	2.0
Condom use during sexual intercourse		
Yes	205	51.0
No	197	49.0
Use of protective gears		
Yes	363	90.3
No	39	9.7
Frequency of protective gear use		
Daily, so long as being at work	298	74.1
Once in a while	65	16.2
Depends on availability	39	9.7

### 3.3. Uptake of hepatitis B vaccine among HCWs

Among all the interviewed HCWs, only 18.9% (76 /402) were fully vaccinated. Most of those who had not received the HBV vaccine 55% (100/182) claimed that the availability of the vaccine at their working facilities was the main hindrance. Table 3 shows the vaccination status and the identified barriers.

**Table 3:** Vaccination status of HCWs and barriers to vaccination (*N* = 402).

Variable	Frequency (*n*)	Percentage (%)
HBV vaccination status		
Vaccinated with at least one shot	220	54.7
Never been vaccinated	182	45.3
Number of shots received		
Never vaccinated	182	45.3
One	45	11.2
Two	99	24.6
Three	76	18.9
Reasons for not vaccinating		
Unavailability of vaccine at my facility	100	54.9
I have never thought of getting the HBV vaccine	33	18.1
I do not know where to find the HBV vaccine	4	2.2
I have not heard of the HBV vaccine	6	3.3
No enough motivation	18	9.9
No money to cover the cost	7	3.8
Not ready to be vaccinated	5	2.7
Other reasons	9	4.9

### 3.4. Comparison of vaccination status among HCWs in Ilemela and Misungwi districts

**Figure 1:**
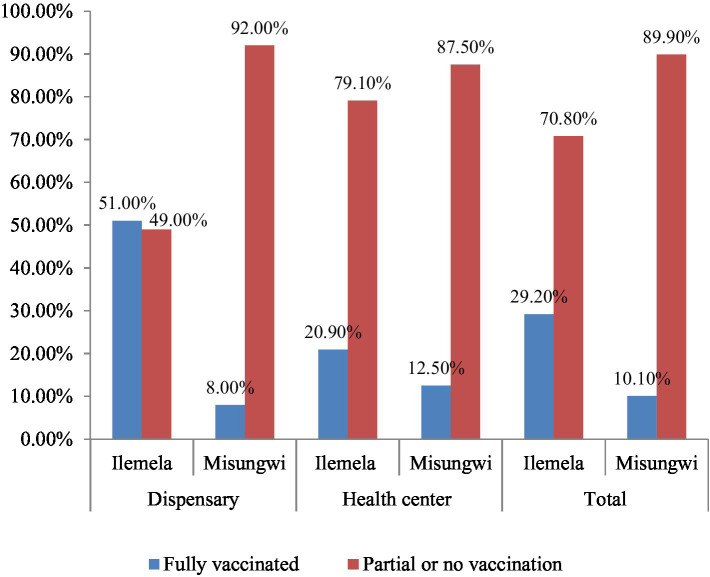
Comparison of vaccination status among HCWs in Ilemela and Misungwi districts by facility levels.

HCWs in the Ilemela district were more likely to be fully vaccinated as compared to their counterparts in Misungwi (29.2% vs. 10.1%; aOR 5.75, 95% CI 2.91–11.35, *p* = 0.01). Generally, healthcare workers working in dispensaries showed higher hepatitis B vaccine completion compared to those working in health centers in both study areas, at 21.35% versus 17.2% (aOR 0.77, 95% CI 0.46–1.27, *p* > 0.05). Table 4 and Figure 1 show the details of the above description.

**Table 4:** Comparison of vaccination status among HCWs in Ilemela and Misungwi districts (*N* = 402).

District	Fully Vaccinated *n* (%)	Not vaccinated and partial vaccination n (%)	aOR value	value of *p*
Ilemela	54 (29.2%)	131(70.8%)		
Misungwi	22 (10.1%)	195 (89.9%)	5.75 (2.91–11.35)	<0.01

### 3.5. Factors associated with HBV vaccine uptake among HCWs

During the multivariate logistic regression analysis, a number of the factors showed significant association with full vaccination, including the participants’ sex, district, duration of work, history of needle prick injury, and high perceived susceptibility to HBV infection. Male participants had 2.38 higher odds of being vaccinated (aOR = 2.38, 95% CI 1.28–0.45, *p* = 0.006) than to female participants. HCWs in the Ilemela district were five times more likely to be vaccinated (aOR = 5.75, 95% CI 2.91–11.35, *p* < 0.01) than HCWs in Misungwi, and HCWs who had worked for more than 2 years had 3.58 higher odds of being vaccinated (aOR-3.58 95% CI 1.19–10.74, *p* = 0.023) than those who had worked for less than 2 years. On top of that, the participants who had a high perception of susceptibility to HBV infection had 2.20 higher odds of being vaccinated (aOR = 2.20, 95% CI1.02–4.75, *p* = 0.044) than those who had low perception. In addition, HCWs with a history of needle prick injuries were six times more likely to be vaccinated (aOR = 6.87, 95%CI 3.55–13.26, *p* < 0.01) than those who had never encountered accidental needle stick injuries. Details of the above description are tabulated below (Table 5).

**Table 5:** Factors associated with full HBV vaccine uptake among HCWs in Misungwi and Ilemela districts (*n* = 402).

Variable	Fully vaccination	Partial or no vaccination	Crude OR (95% CI)	value of p (95%CI)	Adjusted OR	value of *p* (95% CI)
Age						
15–44 years	69 (19.8%)	280 (80.2%)	Reference			
45 years and above	7 (13.2%)	46 (86.8%)	0.62 (0.27–1.43)	0.26		
Sex						
Female	38 (16.7%)	189 (83.3%)	Reference			
Male	38 (21.7%)	137 (78.3%)	1.38 (0.84–2.28)	0.20	2.38 (1.28–4.45)	0.006
District						
Misungwi	22 (10.1%)	195 (89.9%)	Reference			
Ilemela	54 (29.2%)	131 (70.8%)	3.65 (2.12–6.29)	<0.01	5.75(2.91–11.35)	<0.01
Facility level						
Dispensary	35 (21.3%)	129 (78.7%)	Reference			
Health center	41 (17.2%)	197 (82.8%)	0.77 (0.46–1.27)	0.30		
Years of employment						
Less than 2 years	5 (9.8%)	46 (90.2%)	Reference			
More than 2 years	71 (202%)	280 (79.8%)	2.33 (0.89–6.09)	0.076	3.58(1.19–10.74)	0.023
Medical cadre						
Medical attendant	10 (12.7%)	69 (87.3%)	Reference			
Nurse	24 (16.2%)	124 (83.8%)	0.49 (2.36–10.62)	<0.01	0.09 (0.36–0.25)	<0.01
Laboratory personnel	14 (31.8%)	30 (68.2%)	0.42 (2.07–7.94)	<0.01	0.16 (0.05–0.29)	<0.01
pharmacist	1 (8.3%)	11 (91.7%)	0.48 (1.19–3.30)	0.013	0.30 (0.12–0.78)	0.013
Clinical officer	7 (8.1%)	79 (91.9%)	1.10 (2.83–16.92)	<0.01	0.06 (0.02–0.51)	0.01
Medical doctor	20 (60.6%)	13 (39.4%)	0.53 (2.85–17.36)	0.01	0.06 (0.02–0.16)	<0.01
Needle prick injuries						
No	19 (8.9%)	195 (91.1%)	Reference			
Yes	57 (30.3%)	131 (69.7%)	4.47 (2.54–7.85)	<0.01	6.87(3.55–13.26)	<0.01
Perceived susceptibility						
Low susceptibility	15 (9.3%)	146 (90.7%)	Reference			
High susceptibility	61 (25.3%)	180 (74.7%)	3.30 (1.80–6.04)	<0.01	2.20 (1.02–4.75)	0.044
Perceived severity						
Low severity	73 (19.0%)	311 (81.0%)	Reference			
High severity	3 (16.7%)	15 (83.3%)	0.85 (0.24–3.02)	0.80		
Perceived benefits						
Low benefits	74 (21.0%)	279 (79.0%)	Reference			
High benefits	2 (4.1%)	47 (95.5%)	0.16 (0.04–0.68)	0.005	0.34 (0.07–1.53)	0.159
Perceived efficacy						
Low efficacy	28 (13.2%)	184 (86.8%)	Reference			
High efficacy	48 (25.3%)	142 (74.7%)	2.22 (1.33–3.12)	0.002	0.90 (0.44–1.84)	0.774
Barriers to action						
high barriers	35 (15.0%)	199 (85.0%)	Reference			
Low barriers	41 (24.4%)	127 (75.6%)	1.84 (1.11–3.04)	0.017	1.08 (0.58–2.03)	0.80
Cues to action						
Less motivated to vaccinate	32 (12.5%)	225 (87.5%)	Reference			
Highly motivated to vaccinate	44 (30.3%)	101 (69.7%)	3.06 (1.84–5.11)	<0.00	1.81 (0.92–3.58)	0.087
Knowledge						
Poor and average knowledge	41 (18.1%)	186 (81.9%)	Reference			
Good knowledge	35 (20.0%)	140 (80.0%)	1.13 (0.69–1.87)	0.62		

## 4. Discussion

Hospital-acquired infections contribute to a significant loss of human resources for health and affect the quality of services. HBV infection is prominent among healthcare workers across the globe despite being a vaccine-preventable disease. In Tanzania, studies of the uptake of the HBV vaccine have focused on healthcare workers in tertiary hospitals, and little is known about those working in primary facilities. The aim of this study was therefore to determine the extent to which HCWs in primary health facilities are vaccinated against HBV and the associated factors.

This study revealed that less than 20% of HCWs received full vaccination, leaving the rest with no or partial vaccination. These findings are different from studies at tertiary hospitals in Tanzania, where the completion rate has been demonstrated to be higher, ranging from 33 to 70% (7, 18, 19). The difference in uptake could be attributed to vaccine availability, because in high-level facilities the HBV vaccine is usually available and sometimes free of charge (19). On the other hand, the HBV vaccine is not readily available at most primary health facilities in Tanzania, as evidenced by this study. Therefore, the Ministry of Health must consider the HBV vaccine supply throughout lower and higher levels of facilities to increase uptake in these disadvantaged facilities. However, these findings are quite similar to those provided by a study conducted in Somalia, where it was found that only 56% of HCWs had received the HBV vaccine, with a very low completion rate of 16.6% (20). This shows that HCWs need to be sensitized more on vaccine completion, because most of them showed higher non-completion status.

In this study, only 43.5% of HCWs had good knowledge of HBV infection. It is therefore important that HCWs are educated about occupational diseases, in particular HBV, to increase their hepatitis B vaccine uptake, as studies have shown a significant association between good knowledge and HBV vaccine uptake (19, 21).

The occupational risk of acquiring HBV infection was also explored in this study, and it was found that splashes of body fluids from patients were the most common occupational accidents at an occurrence of 83.1%, followed by needle stick injuries at 46.8%. The incidences of body fluid splashes are higher than that found in a study in Cameroon, where only 56% of participants reported similar accidents (22). This shows that HCWs are at risk of acquiring infections, given the risk of needle stick injuries and body fluid splashes, which might be infected and expose them to infection acquisition.

The factors that were found to be significantly associated with HBV vaccine uptake include the sex of the participant, area of residence, duration of employment, history of needle prick injury, and high perception of susceptibility to HBV infection.

Residents of the Ilemela district showed higher rates of vaccine completion compared to HCWs in Misungwi, which represents the rural setting of the study. The findings exemplify studies in other regions of Africa, where urban residents showed higher HBV vaccine uptake compared to rural residents (23, 24). This is because there is limited research and supplies in rural settings compared to urban settings, which could be a cause of this compromise. Therefore, a focus should also be centered on rural health facilities to protect this vulnerable group.

Another factor that showed a significant association with vaccination was a history of needle stick injuries. Needle stick injuries are very common among HCWs; most studies report a prevalence range between 40 and 65% (5, 6, 25). This predisposes them to the risk of acquiring HBV infection and therefore the need for vaccination to prevent infection acquisition. Therefore, this finding emphasizes the need for sensitization and resource mobilization campaigns among HCWs on infection prevention control strategies to reduce the risk of acquiring HBV and other blood born infections. This will help increase their vaccination rates and reduce their risk of acquiring HBV infection through occupational accidents.

Male respondents were more likely to uptake full vaccination than female participants in this study. This finding is incongruent with other studies conducted elsewhere in Africa, most of which reported higher vaccination coverage among female participants (26–28). This was related to the fact that female people are usually more conscious when it comes to disease prevention and control (26). The implication of this finding in our study is that male people have now become more responsive to their health and are willing to take part in the whole process of disease prevention.

Duration of employment was significantly associated with full vaccination status among the participants. The participants who had worked for more than 2 years were more likely to have complete vaccination than those who had less than 2 years of employment in this study. The findings are in line with the findings in the Muhimbili national hospital, where employees who had a long duration of employment were likely to have been vaccinated fully (7). Moreover, studies conducted in Ghana and Uganda among medical students showed vaccination to be associated with advanced years of study among participants (28, 29). The cause of this may be an increased understanding of HBV disease with years of work and study that drive HCWs and students to receive vaccines.

Moreover, HCWs who perceived higher susceptibility to HBV infection showed a significant HBV vaccination rate. This is probably a good sign, as this shows that with a higher perception of infection the prevention strategies are likely to be taken into account, as evidenced by the study participants; however, the vaccination rate is not yet satisfactory. The findings are in line with a study conducted among HCWs in Ethiopia, which showed a significant vaccination rate with the perception of higher susceptibility to HBV infection (6, 26). However, this finding is different to most other studies that showed that participants who perceived higher HBV infection susceptibility still had low vaccination rates (5, 6, 25, 26). Therefore, national programs should emphasize vaccination, as it is possible to vaccinate with a higher perception of infection acquisition.

Most of the studies only focus on only disease status and screening, but not on the linkage to HBV vaccination centers, of which this study is an example. Therefore, it is important to have more studies that screen, provide free vaccines to, and link the diseased HCWs to treatment clinics for follow-up. The studies and campaigns should be conducted at least twice a year to capture HCWs who are newly employed, as this study showed that those who had worked for less than 2 years were unlikely to have received the HBV vaccine. This will help to increase coverage of HBV vaccination and hence reduce HCW–patient transmission of HBV. Moreover, it is important to have HBV vaccination as a compulsory requirement before employment to increase vaccine uptake among newly employed HCWs.

## 5. Conclusion

This study showed low coverage of the hepatitis B vaccine among healthcare workers despite the existence of risky exposures. Therefore, advocacy campaigns and HBV vaccine mobilization in primary health facilities are pivotal to saving this important population group. Moreover, there is a need for continuous medical training on infection prevention and control. The HBV vaccine should be forwarded as one of the prerequisites for employment among HCWs to protect them from the risk of acquiring HBV infection during their practice. Further studies on seroprevalence are recommended among HCWs in primary levels of facilities to attain reliable data on the prevalence of HBV infection among HCWs in these facilities and HBV vaccine uptake.

### 5.1. Limitations of the study and recommendations

This study used only questionnaires to assess vaccine uptake and HBV status among HCWs. It did not go further to assess HBsAg or HBsAb status among HCWs, which are measures of HBV infection status and exposure status to HBV. Furthermore, vaccination status was self-reported among respondents, and there was no available documentation to verify that they were vaccinated. It is therefore important that as vaccination programs continue operating documentation should be given to the clients to have proof of vaccination against HBV. Moreover, further studies should be conducted that assess the vaccination status among these HCWs through the measurement of HBV antibodies acquired through vaccination to attain more reliable data on seroprevalence.

## Data availability statement

The raw data supporting the conclusions of this article will be made available by the authors, without undue reservation.

## Ethics statement

The studies involving human participants were reviewed and approved by the Joint Catholic University of Health and Allied Sciences–Bugando Research and Ethics Committee (CREC/573/2022). The participants provided their written informed consent to participate in this study.

## Author contributions

BN, FM, and AK designed the study and conception. BN, FH, and DW conducted and supervised data collection, data analysis, and interpretation of the findings. BN and DW wrote the first draft of the manuscript. SK, FM, MM, and AK critically reviewed the manuscript and approved its submission. All authors approved the submission of the manuscript.

## Conflict of interest

The authors declare that the research was conducted in the absence of any commercial or financial relationships that could be construed as a potential conflict of interest.

## Publisher’s note

All claims expressed in this article are solely those of the authors and do not necessarily represent those of their affiliated organizations, or those of the publisher, the editors and the reviewers. Any product that may be evaluated in this article, or claim that may be made by its manufacturer, is not guaranteed or endorsed by the publisher.
